# Changes in soluble tumor necrosis factor receptor type 1 levels and early renal function decline in patients with diabetes

**DOI:** 10.1111/jdi.13061

**Published:** 2019-06-03

**Authors:** Richard J MacIsaac, Matthew Farag, Varuni Obeyesekere, Michele Clarke, Ray Boston, Glenn M Ward, George Jerums, Elif I Ekinci

**Affiliations:** ^1^ Department of Endocrinology and Diabetes St Vincent's Hospital Melbourne Melbourne Victoria Australia; ^2^ Department of Medicine University of Melbourne Melbourne Victoria Australia; ^3^ Endocrine Center Austin Health Melbourne Victoria Australia; ^4^ Clinical Chemistry St Vincent's Hospital Melbourne Melbourne Victoria Australia

**Keywords:** Diabetes, Nephropathy, TNF receptors

## Abstract

The relationship between serial changes in soluble tumor necrosis factor receptor type 1 (TNFR1) levels and an early decline in estimated glomerular filtration rate (eGFR) decline remains to be defined. We found that in patients with an early decline in renal function (*n* = 30), soluble TNFR1 values increased (2,595 ± 683 vs 3,596 ± 1,203 pg/mL,* P* < 0.001) as eGFR decreased (89 ± 1 vs 51 ± 2 mL/min/1.73m^2^, *P* < 0.001) over an 8‐year period. In contrast, there were no significant changes in soluble TNFR1 levels in patients with stable renal function (*n* = 17). In a multilevel mixed effects regression model, changes in soluble TNFR1 levels were found to be independently associated with eGFR decline (*Z* = −4.31, *P* < 0.001). An early decline in eGFR is associated with an increase in soluble TNFR levels; however, the factors driving this increase and the possible pathological role that soluble TNFR1 plays in progressive diabetic kidney disease remain to be determined.

## Introduction

Low‐grade chronic inflammation is increasingly recognized as a major driver for the development and progression of diabetic kidney disease (DKD)^1^, ^2^, ^3^, ^4^. Tumor necrosis factor (TNF) is a key mediator of inflammation and plays a role in apoptosis. TNF mediates its signal through two distinct receptors, TNF receptor 1 (TNFR1) and TNF receptor 2 (TNFR2), which are membrane‐bound and also present in a soluble form in serum^5^. Baseline serum levels of soluble TNFRs (sTNFRs) are linked to the progression of DKD, and might have a stronger prognostic ability for the development of end‐stage renal disease than albuminuria^6^. Furthermore, a recent study has shown that baseline sTNFR levels are independently associated with a higher risk of estimated glomerular filtration rate (eGFR) decline in the setting of early or advanced DKD^7^. The ability of baseline levels of sTNFRs to show an exaggerated risk for DKD might be enhanced by considering longitudinal patterns of the levels of the receptor(s)^8^. Therefore, the aim of the present pilot study was to compare changes in sTNFR1 levels in patients with stable or an early decline in renal function.

## Methods

The participants involved in this study attended diabetes clinics at Austin Health, a University of Melbourne tertiary referral center, in Melbourne, Australia. From a clinical database, we identified 47 patients with either type 1 or 2 diabetes that had at least four estimations of GFR over a 4‐year period (with minimum time between eGFR measurements of 4.7 months) and an initial eGFR >60 mL/min/1.73 m^2^. Patients were then divided into two groups on the basis of their change in renal function; that is, those with stable or an early decline in renal function. Patients were considered to have an early decline in renal function if their rate of eGFR decline was >3.5 mL/min/1.73 m^2^ per year with a final eGFR <60 mL/min/1.73 m^2^ 9.

Clinical and biochemical assessments were made at four time intervals for each patient (mean time with the follow up for stable and early renal function decline patients was not different, 7.4 [interquartile range 6–8] years and 7.7 [interquartile range 6–9] years, respectively). GFR was estimated using the creatinine Chronic Kidney Disease Epidemiology Collaboration formula^10^. Written informed consent was obtained from participants in this study for the unrestricted use of their clinical data for non‐interventional research studies, as approved by the Austin Health Human Research Ethics Committee.

We measured sTNFR1 levels in stored serum samples using an enzyme‐linked immunoassay kit (Human sTNFR1 EIA‐ BIO 94) obtained from EKF diagnostics (Dublin, Ireland). Patient serum was retrieved from frozen samples, and stored from 2001 to 2015. The coefficients of variation for intra/interassay precision (as assessed by the manufacturer) were 3.3 and 5.1%, respectively, and as described previously^11^.

Group differences at baseline were compared using *t*‐tests and non‐parametric tests where appropriate. Analysis of variance (anova) was used to analyze the differences among group means for sTNFR1 levels and eGFR across time, with the Tukey–Kramer test used to make pairwise comparisons within the two groups of patients. Multilevel mixed‐effects regression models were used to examine the relationship between changes in biochemical/clinical variables and eGFR across time.

## Results

The initial clinical and biochemical parameters for patients with stable and an early decline in renal function are shown in Table 1. Both groups of patients were matched for baseline parameters apart from age. By definition, eGFR values progressively decreased during the follow‐up period (*F* = 90, *P* < 0.001) in the early declining group, with this decrease being accompanied by a significant increase in sTNFR1 values (*F* = 90.0, *P* < 0.001). In the early eGFR declining group, a significant increase in sTNFR1 levels was already apparent after 2–4 years of follow up (change in levels: 660 pg/mL, *P* < 0.05). The rate of change in sTNFR1 levels over the entire follow‐up period also correlated with the rate of change in eGFR, but only patients with an early decline in renal function (*n* = 30) were considered (*r* = −0.45, *P* < 0.05, data not shown).

**Table 1:** Baseline clinical characteristics and biochemical variables for patients with stable or an early decline in renal function

Variable	Stable renal function (*n* = 17)	Early decline in renal function (*n* = 30)	*P*‐value
Median diabetes duration (years)	31.0 [19–35]	23.5 [19–21]	0.10
Diabetes type (% type 2)	65	87	0.08
Median age (years)	58 [49–61]	64 [58–72]	0.009
Median follow up (years)	7.41 [6–8]	7.73 [6–9]	0.94
Sex (% male)	59	60	0.94
Retinopathy (%)	47	57	0.53
ACE inhibitor use (%)	59	47	0.42
ARB use (%)	35	63	0.06
Statin use (%)	65	87	0.08
Fenofibrate use (%)	6	7	0.92
Insulin use (%)	71	67	0.78
BMI (kg/m^2^)	30.3 [28–34]	28.5 [26–33]	0.08
eGFR (mL/min/1.73 m^2^)	95 ± 17	89 ± 11	0.15
AER (μg/min)	7.60 [5–11]	19.25 [11–95]	0.06
Soluble TNFR1 (pg/mL)	2,560 ± 693	2,595 ± 683	0.87
HbA1c (%)	7.66 ± 0.83	8.04 ± 1.31	0.29
HbA1c (mmol/mol)	61 ± 9	64 ± 14	0.29
SBP (mmHg)	133 ± 7	130 ± 11	0.31
Total cholesterol (mmol/L)	4.78 ± 0.98	4.59 ± 0.97	0.52
Triglycerides (mmol/L)	1.70 [0.9–2.4]	2.00 [1.5–2.5]	0.33

Data presented as the median [interquartile range] or mean ± standard deviation. Values are at baseline unless otherwise stated. ACE, angiotensin‐converting enzyme; AER, albumin excretion rate; ARB, angiotensin receptor blocker; BMI, body mass index; eGFR, estimated glomerular filtration rate; HbA1c, glycated hemoglobin 1c; SBP, systolic blood pressure; TNFR1, tumor necrosis factor receptor type 1.

**Figure 1:**
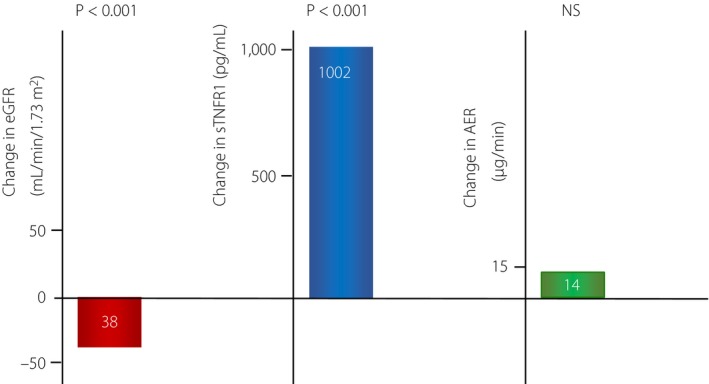
Changes in estimated glomerular filtration rate (eGFR), soluble tumor necrosis factor receptor type 1 (TNFR1) and albumin excretion rate (AER) from baseline in patients with an early decline in renal function (*n* = 30) after 8 years of follow up. *P*‐values compare change of the relative variable with the change observed in patients with stable renal function.

For patients with an early decline in renal function, eGFR decreased (89 ± 1 vs 51 ± 2 mL/min/1.73 m^2^, *P* < 0.001) and sTNFR1 values increased (2,595 ± 683 vs 3,596 ± 1,203 pg/mL, *P* < 0.001) from baseline to the end of the follow‐up period, respectively. There were no significant changes in the albumin excretion rate (AER) in the early declining renal function group. The change from baseline to the end of follow up for eGFR, sTNFR1 levels and AER are shown in Figure**** 1. There were no significant changes in sTNFR1 levels or AER in the stable renal function group. Although some parameters changed during the study period within each group, there were no significant differences in any final clinical or biochemical variable for stable or early declining renal function patients apart from sTNFR1 values and eGFR (Table 2).

**Table 2:** Initial and final clinical and biochemical variables for patients with stable or an early decline in renal function

Variable	Stable renal function (*n* = 17)			Early decline in renal function (*n* = 30)			(*n* = 30) Difference between final values (*P*‐ value)
	Initial (mean)	Final (mean)	*P*‐value	Initial (mean)	Final (mean)	*P*‐value	
Soluble TNFR1 (pg/mL)	2,560 ± 693	2,374 ± 766	0.37	2,595 ± 683	3,597 ± 1203	<0.0001	<0.0001
eGFR (mL/min/1.73 m^2^)	95 ± 17	96 ± 15	0.44	89 ± 11	51 ± 15	<0.0001	0.0005
AER (μg/min)	7.60 [5–11]	9.20 [5–20]	0.28	19.25 [11–95]	33.10 [8–228]	0.0645	0.087
HbA1c (%)	7.66 ± 0.83	7.77 ± 0.95	0.61	8.04 ± 1.31	7.73 ± 1.16	0.27	0.9107
HbA1c (mmol/mol)	60.2 ± 6.52	61.4 ± 7.51	0.61	64.4 ± 10.50	61.0 ± 9.15	0.27	0.9107
SBP (mmHg)	133 ± 7	133 ± 11	0.91	130 ± 11	135 ± 16	0.03	0.5671
Total cholesterol (mmol/L)	4.78 ± 0.98	3.89 ± 0.85	0.01	4.59 ± 0.97	3.87 ± 1.07	0.001	0.9281
Triglycerides (mmol/L)	1.70 [0.9–2.4]	1.30 [0.9–1.5]	0.52	2.00 [1.5–2.5]	1.55 [0.9–2.0]	0.0026	0.201
BMI (kg/m^2^)	30.30 [28–34]	32.00 [29–34]	0.0343	28.00 [25–30]	29.50 [26–33]	0.0011	0.245

Data presented as the median [interquartile range] or mean ± standard deviation. AER, albumin excretion rate; BMI, body mass index; eGFR, estimated glomerular filtration rate; HbA1c, glycated hemoglobin 1c; SBP, systolic blood pressure; TNFR1, tumor necrosis factor receptor type 1.

The results of multilevel mixed‐effects regression models for various clinical and biochemical variables with changes in eGFR as the dependent variable are shown in Table 3. In model 1, which was adjusted for changes in AER, changes in sTNFR1 levels were independently associated with eGFR decline (*Z* = −3.37, *P* < 0.01) over the follow‐up period of the study. By definition, follow‐up time was also significantly associated with the decline in eGFR in all of the regression models. In model 2, which was also adjusted for age, sex and glycated hemoglobin, as well as changes in AER, TNFR1 levels remained independently associated with eGFR decline (*Z* = −3.49, *P* < 0.001). Furthermore, in a fully adjusted model that included 18 clinical and biochemical variables with changes in eGFR as the dependent, changes in sTNFR1 levels still remained independently associated with eGFR decline (*Z* = −4.31, *P* < 0.001).

**Table 3:** Results of a multilevel mixed‐effects regression analysis examining the relationship between clinical characteristics and biochemical variables with changes in estimated glomerular filtration rate

Independent variables	Coefficient	SE	*Z*‐value	95% Confidence interval	*P*‐value
Model 1					
Soluble TNFR1 (pg/mL)	−0.004	0.001	−3.37	−0.006 to −0.002	<0.01
Follow‐up time (years)	−6.58	0.97	−8.82	−8.475 to −4.691	<0.001
AER (μg/min)	−0.026	0.003	−0.94	−0.082 to 0.003	0.346
Model 2					
Soluble TNFR1 (pg/mL)	−0.004	0.001	−3.49	−0.006 to −0.017	<0.001
Follow‐up time (years)	−6.50	0.962	−6.76	−8.390 to −4.619	<0.001
AER (μg/min)	−0.027	0.003	−0.90	−0.008 to 0.003	0.366
Male (yes/no)	−2.62	4.250	−0.62	−10.954 to 5.705	0.537
Age (years)	−0.535	0.189	−2.82	−0.907 to −0.166	<0.01
Model 3					
Soluble TNFR1 (pg/mL)	−0.005	0.001	−4.31	−0.007 to −0.003	<0.001
Follow‐up time (years)	−6.017	1.013	−5.94	−8.002 to −4.032	<0.001
AER (μg/min)	−0.002	0.003	−0.80	−0.008 to 0.003	0.423
Male (yes/no)	−2.591	4.061	−0.64	−10.551 to 5.369	0.524
Age (years)	−0.822	0.243	−3.38	−1.299 to −0.345	0.001
Diabetes duration (years)	0.501	0.238	2.10	0.034 to 0.968	0.036
Type 2 diabetes (yes/no)	7.654	6.353	1.20	−4.799 to 20.106	0.228
Insulin use (yes/no)	−1.284	4.649	−0.28	−10.396– 7.827	0.782
ARB use (yes/no)	−7.959	4.468	−1.78	−16.716 to 0.799	0.075
ACE inhibitor use (yes/no)	4.797	4.749	1.01	−4.511 to 14.105	0.312
Statin use (yes/no)	−1.465	6.090	−0.24	−13.401 to 10.470	0.81
Retinopathy (yes/no)	−2.808	4.915	−0.57	−12.441 to 6.825	0.568
HbA1c (%)	−0.0414	0.820	−0.05	−1.649 to 1.566	0.96
Total cholesterol (mmol/L)	1.770	1.159	1.53	−0.502 to 4.042	0.127
Triglycerides (mmol/L)	−0.115	1.034	−0.11	−2.142 to 1.911	0.911
BMI (m^2^/kg)	0.330	0.402	0.82	−0.457 to 1.118	0.411
SBP (mmHg)	−0.074	0.090	−0.82	−0.249 to 0 0.102	0.411
DBP (mmHg)	0.163	0.117	1.39	−0.067 to 0.393	0.164

Baseline variables included in the model included sex (male), age, diabetes duration, type of diabetes (type 2 vs type 1), insulin use, angiotensin receptor blocker (ARB) use, angiotensin‐converting enzyme (ACE) inhibitor use, statin use and presence of retinopathy (yes/no). Changes in soluble tumor necrosis factor receptor type 1 (TNFR1), albumin excretion rate (AER), follow‐up time points, total cholesterol, triglycerides, body mass index (BMI), systolic and diastolic blood pressure (BP) during the follow‐up period of the study were also included in the model. Model 1 was adjusted for AER (model performance: χ^2^ = 75.1, *P* < 0.001). Model 2 was adjusted for AER, age, sex and glycated hemoglobin (HbA1c; model performance: χ^2^ = 86.1, *P* < 0.001). Model 3 was fully adjusted for all variables measured (model performance: χ^2^ = 120.6, *P* < 0.001). DBP, diastolic blood pressure; eGFR, estimated glomerular filtration rate; SBP, systolic blood pressure.

The only other variables independently related to changes in eGFR in the fully adjusted model included follow‐up time, patient's age at the start of the study and diabetes duration. Furthermore, when the fully adjusted model was constructed with and without the addition of changes in sTNFR1 levels, it was found that including the changes in sTNFR1 levels significantly increased the model's ability to predict the risk for an early decline in renal function (χ^2^ likelihood ratio = 15.2, *P* = 0.0001).

## Discussion

The present small pilot study suggests that there is a temporal relationship between an increase in sTNFR1 levels and an early decline in eGFR. This relationship is independent of other factors, such as changes in albuminuria, glycated hemoglobin and systolic blood pressure. Changes in sTNFR1 levels also improved prediction for renal function decline. Monitoring changes in sTNFR1 levels might therefore emerge as a promising biomarker‐based method for evaluating an individual patient's risk for the development and progression of DKD. It could also prove useful for gauging the effectiveness of novel approaches that aim to slow renal function loss. However, given our small sample size and the fact that urinary clearance of TNF receptors was not assessed, the results of this pilot study should only be interpreted as hypothesis generating.

The molecular weight of sTNFR1 is just 55 KD, and despite some presumed ectodomain cleavage of the receptor, is the predominant form of sTNFR1 found in human sera^12^. A contribution of plasma accumulation of the receptor as GFR declines through reduced renal clearance is therefore possible and, indeed, sTNFR1 levels are detectable in urine, and serum levels have been reported to correlate with eGFR in cross‐sectional studies^13^, ^14^, ^15^. However, the present results could also be consistent with the growing body of evidence implicating sTNFR in the pathogenic processes that promote GFR loss in diabetes^6^, ^16^, ^17^. Currently, the exact role that TNF and its soluble receptors play in DKD remains unknown. However, inflammatory processes, reflected by activation of the TNF system, have been consistently linked to the development and progression of DKD^6^, ^18^, ^19^.

Thresholds for baseline levels or rates of changes of sTNFR levels over time that indicate an increased risk for DKD have yet to be established. Indeed, we did not detect a difference between baseline sTNFR1 levels between patients with subsequent stable or declining eGFR levels, as seen in larger studies^20^, ^21^, ^22^, ^23^. However, given the relatively small number of patients with stable renal function that we studied, and the fact that both groups of patients were very well matched for baseline clinical and biochemical parameters, our finding of similar initial sTNFR1 levels between the two groups of patients with different GFR trajectories might not be an entirely unexpected finding.

To date, there has only been a very limited assessment of possible changes in sTNFR levels over time in relation to parameters related to renal function. In a study from the Joslin Diabetes Center in the USA, the sTNFR1 levels of 77 patients type 1 diabetes mellitus were measured at baseline and after 2.5 years of follow up^21^. These patients were presumably randomly selected from a larger group of 625 patients in which higher baseline sTNFR1 levels were shown to be associated with a greater chance of achieving stage 3 chronic kidney disease (eGFR <60 mL/min/1.73 m^2^) after 12 years of follow up. In that study, just 69 patients (11%) developed an eGFR <60 mL/min/1.73 m^2^. In the subgroup of patients (*n* = 77) with a repeat sTNFR1 level measurement, no significant change in TNFR1 was found. However, given that many patients who had a repeat sTNFR1 level measurement where not selected on the basis of change in renal function, many of these patients might not have experienced any significant decline in eGFR during the follow‐period. In contrast, in the present patients that were selected for an early decline in eGFR, sTNFR1 levels started to significantly decline after only a 2–4‐year follow‐up period.

In conclusion, the present findings show that monitoring early changes in sTNFR1 levels, regardless of the absolute baseline level of the receptor, might provide another method of detecting a patient's risk for an early decline in renal function.

## Disclosure

The authors declare no conflict of interest.
